# Systemic Delivery of Protein Nanocages Bearing CTT Peptides for Enhanced Imaging of MMP-2 Expression in Metastatic Tumor Models

**DOI:** 10.3390/ijms16010148

**Published:** 2014-12-24

**Authors:** Takahito Kawano, Masaharu Murata, Jing Shu Piao, Sayoko Narahara, Nobuhito Hamano, Jeong-Hun Kang, Makoto Hashizume

**Affiliations:** 1 Innovation Center for Medical Redox Navigation, Kyushu University, 3-1-1 Maidashi, Higashi-ku, Fukuoka 812-8582, Japan; E-Mails: t-kawano@dem.med.kyushu-u.ac.jp (T.K.); narahara@dem.med.kyushu-u.ac.jp (S.N.); mhashi@dem.med.kyushu-u.ac.jp (M.H.); 2 Department of Advanced Medical Initiatives, Kyushu University, 3-1-1 Maidashi, Higashi-ku, Fukuoka 812-8582, Japan; E-Mails: jingshu@dem.med.kyushu-u.ac.jp (J.S.P.); n.hamano@camiku.kyushu-u.ac.jp (N.H.); 3 Faculty of Medical Sciences and Center for Advanced Medical Innovation, Kyushu University, 3-1-1 Maidashi, Higashi-ku, Fukuoka 812-8582, Japan; 4 Division of Biopharmaceutics and Pharmacokinetics, Department of Biomedical Engineering, National Cerebral and Cardiovascular Center Research Institute, 5-7-1 Fujishirodai, Suita, Osaka 565-8565, Japan; E-Mail: jrjhkang@ncvc.go.jp

**Keywords:** matrix metalloproteinase, protein nanocages, CTT peptides, metastatic tumor, *in vivo* fluorescence imaging

## Abstract

Matrix metalloproteinase 2 (MMP-2) in metastatic cancer tissue, which is associated with a poor prognosis, is a potential target for tumor imaging *in vivo*. Here, we describe a metastatic cancer cell-targeted protein nanocage. An MMP-2-binding peptide, termed CTT peptide (CTTHWGFTLC), was conjugated to the surface of a naturally occurring heat shock protein nanocage by genetic modification. The engineered protein nanocages showed a binding affinity for MMP-2 and selective uptake in cancer cells that highly expressed MMP-2 *in vitro*. In near-infrared fluorescence imaging, the nanocages showed specific and significant accumulation in tumor tissue after intravenous injection *in vivo*. These protein nanocages conjugated with CTT peptide could be potentially applied to a noninvasive near-infrared fluorescence detection method for imaging gelatinase activity in metastatic tumors *in vivo*.

## 1. Introduction

Tumor invasion and metastasis define malignancy, which are the major causes of cancer mortality. Metastasis is a complex multi-step process involving detachment of tumor cells from the primary tumor, invasion through the basement membrane, intravasation into the circulatory system, extravasation at a distant site, and outgrowth of a secondary tumor [1]. Matrix metalloproteinases (MMPs) are a family of Zn^2+^-dependent endopeptidases that play important roles in metastatic processes, particularly MMP-2 (gelatinase A, 72-kD type IV collagenase) and MMP-9 (gelatinase B, 92-kD type IV collagenase) [2,3,4,5]. MMPs degrade the basement membrane and extracellular matrix, thus contributing to tissue remodeling and cell migration. Overproduction and unrestrained activity of MMPs have been linked to malignancy in a variety of tumors including brain, colon, lung, bladder, melanoma, and breast. Indeed, enhanced or unregulated expression of MMP-2 is associated with a poor prognosis in cancer patients [6,7]. Therefore, MMP-2 is a potential target for metastatic tumor imaging.

The cyclic peptide CTTHWGFTLC (CTT) containing a His-Trp-Gly-Phe motif has been described as a selective MMP-2 inhibitor that reduces the migration of both human endothelial and tumor cells, and prevents tumor growth and invasion in animal models [8,9]. The CTT peptides are isolated from phage display peptide libraries as specific gelatinase binding peptides and show selective biding property of MMP-2 and MMP-9 but not of several other MMP family members [8]. Several studies have reported derivatization and radiolabeling of CTT with ^125^I and ^111^In for *in vitro* and *in vivo* imaging. Unimpaired inhibition of MMP-2 activity has been demonstrated *in vitro* using derivatized CTT [10,11]. Positron emission tomography of MMP-2 has been performed in a metastatic tumor model *in vivo* using ^64^Cu-DOTA-CTT [12]. Nevertheless, the rapid clearance and short circulation time in the blood because of the low molecular weight of ^64^Cu-DOTA-CTT limits its applications in imaging. One method to improve pharmacokinetic properties is conjugation of peptide ligands to nano-sized particles including polymer micelles, liposomes, dendrimers, and inorganic nanoparticles [13,14,15,16]. CTT peptides enable retention of bioactivity that would otherwise be lost by direct linkage to nanoparticles, which is very important for the development of effective imaging agents to control pharmacokinetics.

**Figure 1:**
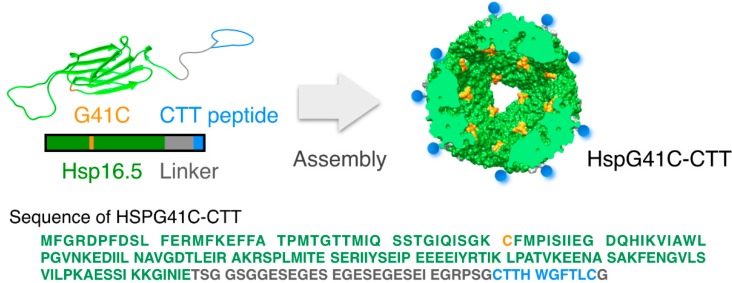
Schematic representation and sequence of the engineered HspG41C-CTT protein nanocage. Blue and gray regions indicate the CTT peptide and hydrophilic linker, respectively. The orange region indicates the Gly residue at position 41, which was mutated to a Cys residue for conjugation to the near-infrared (NIR) fluorophore maleimide.

We focused on development of a metastatic tumor imaging techniques using nanoparticles that specifically target MMP-2 in cancer cells. These nanoparticles were developed by a genetic engineering approach involving the addition of CTT peptides to the exterior surface of the nanoparticle. As a model nanoparticle, we used heat shock protein (Hsp) 16.5, a small naturally occurring protein in *Methanococcus jannaschii* that forms a cage-like structure by self-assembly of 24 subunits. The outer and inner diameters of the cage are 13 and 6.5 nm, respectively [17,18,19]. Hsp nanocages are attractive as a biomedical tool for delivery of imaging agents because of their biocompatibility, monodispersed formation, robust structure, easy acquisition from *Escherichia coli*, and simple functionalization through chemical and genetic strategies [20,21,22,23]. We synthesized engineered protein nanocages that were conjugated with CTT peptides (Figure 1). To link the nanocage to a near-infrared (NIR) fluorescence agent, Gly41 located inside of the native Hsp16.5 nanocage was substituted with Cys. Because NIR fluorescence imaging (650–900 nm) displays properties of low absorption and relatively low autofluorescence, it offers several advantages over other modalities for imaging living organisms. In addition, NIR fluorescence imaging has potentially high spatial resolution, high sensitivity and low tissue damage to the living subject. We determined the physical properties, MMP-2-binding capacity, cytotoxicity, and cellular uptake of CTT peptide-conjugated protein nanocages, and applied them as an NIR fluorescence contrast agent to detect tumor cells *in vivo*.

## 2. Results and Discussion

### 2.1. Characterization of the Protein Nanocages Modified with CTT Peptides

**Figure 2:**
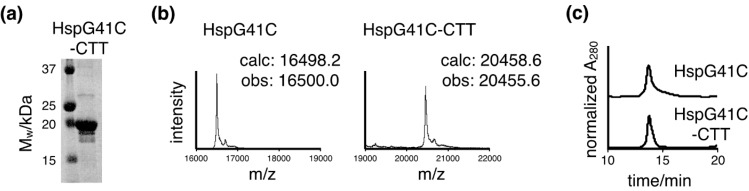
Purification of protein nanocages. (**a**) SDS-PAGE analysis of the recombinant subunits of the nanocages; (**b**) MALDI-ToF mass spectra analysis of the dissociated subunits of HspG41C and HspG41C-CTT. The observed molecular masses are shown; (**c**) Size exclusion chromatography of HspG41C and HspG41C-CTT.

The protein nanocages linked to CTT peptides (HspG41C-CTT) and control nanocages (HspG41C) were prepared using an *E. coli* protein expression system. The HspG41C mutant presents unique reactive cysteine residues on the interior surface of the assembled cage for the attachment of fluorophore molecules, because general fluorophore is hydrophobic and hydrophobic molecules are bound non-specifically *in vivo* [22,23]. The proteins were purified by sequential anion exchange chromatography followed by native size exclusion chromatography. The purified HspG41C-CTT monomer proteins, separated by SDS-polyacrylamide gel electrophoresis (PAGE), appeared as a single band after Coomassie blue staining (Figure 2a). Following dissociation, the molecular weight of the purified nanocages was determined by matrix-assisted laser desorption/ionization time-of-flight (MALDI-ToF) mass spectrometry with a sinapinic acid matrix (Figure 2b). The molecular weights of HspG41C and HspG41C-CTT monomer proteins were determined as 16,500.0 and 20,455.6 Da, respectively, which are in agreement with the corresponding calculated values of 16,498.2 and 20,458.6 Da. Based on the results of size exclusion chromatography, both nanocages were obtained successfully at sufficient purities (Figure 2c).

**Figure 3:**
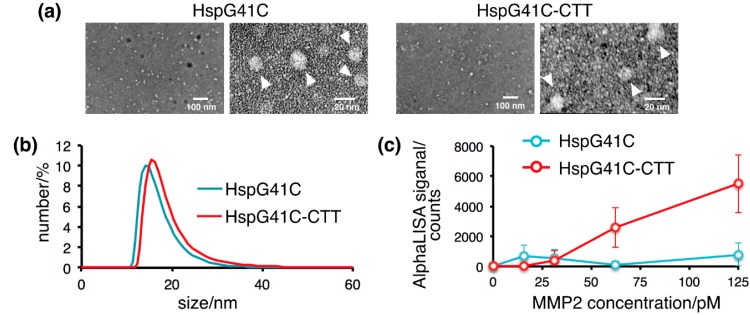
Characterization of the protein nanocages. (**a**) Transmission electron microscopy images of the nanocages. White arrows show the nanocages; (**b**) Dynamic light scattering profiles of the nanocages in phosphate-buffered saline; (**c**) AlphaScreen binding assay of the nanocages with MMP-2.

Transmission electron microscopy (TEM) images of negatively stained HspG41C and HspG41C-CTT confirmed their nanoparticles without large aggregation, regardless of the modification of the exterior surface in HspG41C-CTT (Figure 3a). The sizes of the nanocages under physiological conditions were measured by dynamic light scattering analysis. The mean outer diameters of HspG41C and HspG41C-CTT were 14.1 and 15.5 nm, respectively (Figure 3b). The size distribution peaks of the nanocages shifted toward larger sizes with the addition of CTT peptides to the *C*-terminal region. The nanocages showed a uniform size distribution. In addition, the nanocage structure was maintained in aqueous medium without the formation of large aggregates.

Next, we evaluated the MMP-2-binding property of CTT peptide-modified nanocages. To investigate this interaction, we coupled MMP2 and the nanocages to the AlphaScreen acceptor and donor beads, respectively. HspG41C and MMP-2 interacted poorly, but HspG41C-CTT substantially augmented the MMP2 interaction (Figure 3c). CTT peptides could bind to MMP-2 regardless of linkage to nanocages by hydrophilic linkers.

### 2.2. Cellular Uptake of the Protein Nanocages in Vitro

**Figure 4:**
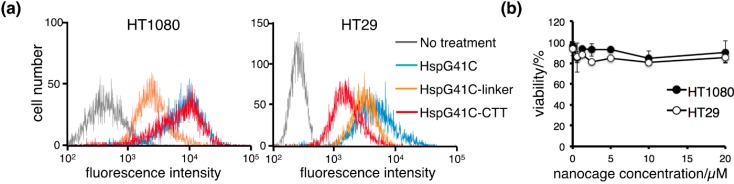
Cellular uptake of the protein nanocages *in vitro*. (**a**) Representative histograms of the fluorescence intensities of HT1080 and HT29 cells incubated with fluorophore-labeled nanocages in the presence of 10% fetal bovine serum for 3 h; (**b**) Cytotoxicity of HspG41C-CTT in HT1080 and HT29 cells. Data are presented as the mean ± standard error of the mean of three independent experiments.

For cellular uptake experiments, Hsp nanocages labeled with a fluorophore (Alexa Fluor 488) were prepared by the Michael addition reaction of Alexa Fluor 488 maleimide and Cys41 (interior of Hsp nanocages). Based on the results of size exclusion chromatography, conjugation with a fluorophore inside of the nanocage did not significantly affect the structure or size of the nanocage. To determine whether HspG41C-CTT nanocages were selectively taken up by MMP-2 targeting, HT1080 and HT29 cells were used as positive and negative controls for MMP-2 expression, respectively. Cellular uptake was observed after 3 h of incubation using a flow cytometer (Figure 4a). Non-targeted HspG41C nanocages were taken up by both cell lines. However, HspG41C-CTT nanocages were taken up by HT1080 cells but not HT29 cells. Cell uptake of HspG41C displaying negatively charged linkers decreased by HT1080 cells due to reduce the non-specific binding. These results showed that the nanocages modified with CTT peptides selectively bound to HT1080 cells that highly expressed MMP-2. MMP-2 is known to localize to the cell surface and cytoplasmic compartment and release into the extracellular space, but is not known to internalize inhibitors upon binding [24,25]. The cellular uptakes of CTT-peptides conjugated imaging agents and liposome were energy dependent active receptor mediated endocytosis, as indicated by the observation of significantly reduced uptake at 4 °C [26,27]. Further studies are needed to clarify the role of MMP-2 in cellular internalization of HspG41C-CTT. Because cytotoxicity is an important factor in selecting materials for the development of imaging carriers, we characterized the effects of the nanocages on cell viability under the same conditions that were used to determine cellular uptake. The results showed no appreciable cytotoxic effects of HspG41C or HspG41C-CTT on HT1080 and HT29 cells (Figure 4b).

### 2.3. Biodistribution of the Protein Nanocages in Vivo

**Figure 5:**
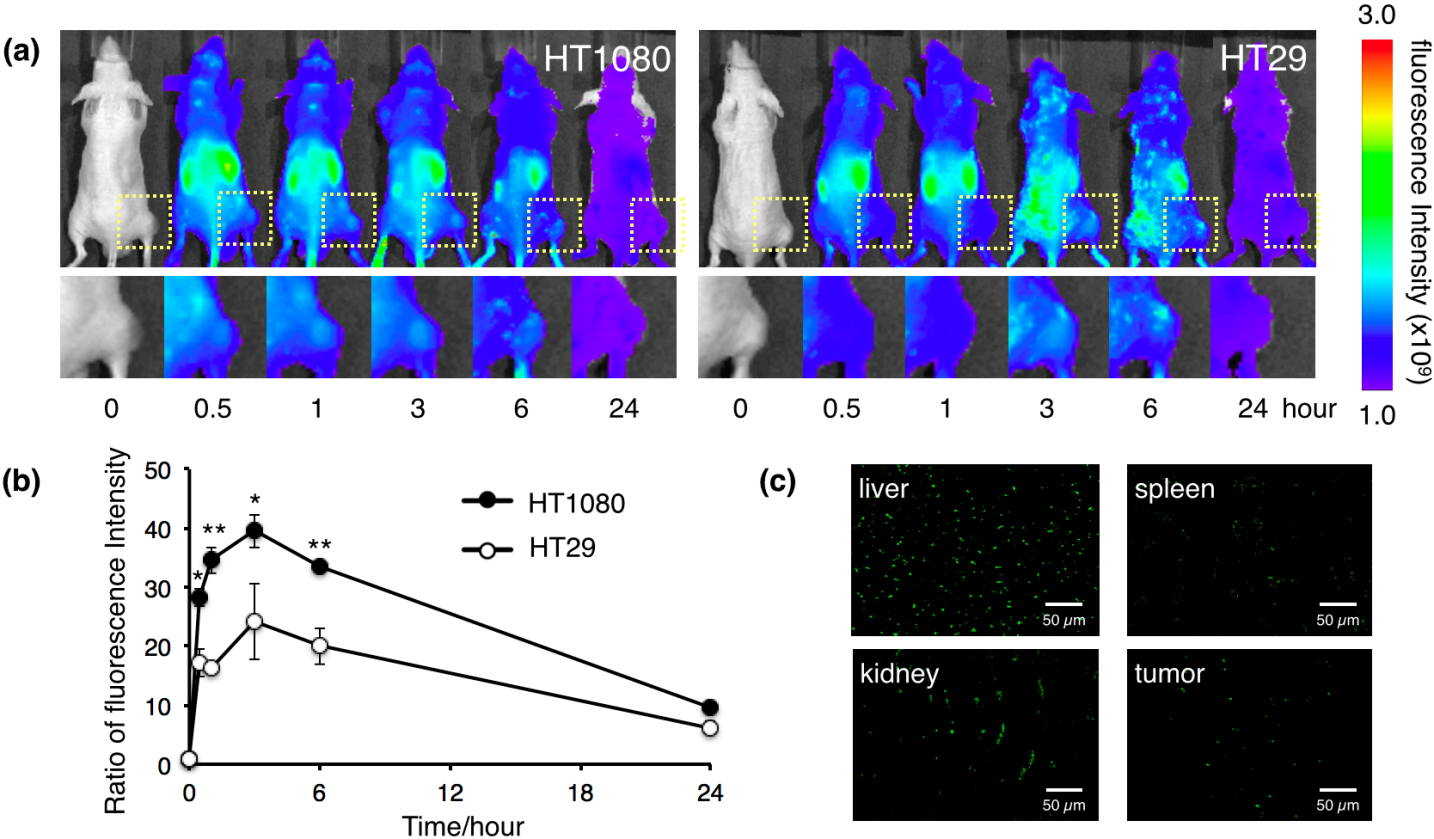
Biodistribution of the protein nanocages *in vivo*. (**a**) *In vivo* real-time NIR fluorescence imaging of intravenously injected Alexa Fluor 750-labeled HspG41C-CTT in HT1080 and HT29 tumor-bearing mice. Time-dependent, tumor-targeting specificities of the nanocages were monitored by the IVIS system. Square regions indicate solid tumor growths of subcutaneously injected cancer cells; (**b**) Fluorescence signal intensity ratio of the tumor/background obtained from *in vivo* images. * *p* < 0.05; ** *p* < 0.01; (c) Organ sections of tumor-bearing mice injected intravenously with Alexa Fluor 488-labeled HspG41C-CTT at 3 h post-injection.

Targeting tumor cells or tumor vasculature by peptides is a promising strategy to deliver therapeutic drugs and imaging agents for cancer therapy. CTT peptide-modified protein nanocages showed specific binding to cancer cells that expressed high levels of MMP-2 *in vitro*. To obtain *in vivo* evidences of tumor targeting, we examined the *in vivo* biodistribution of Alexa Fluor 750-labeled HspG41C-CTT nanocages in tumor-bearing mice. HT1080 and HT29 cells were used as positive and negative controls for MMP-2 expression, respectively. The NIR light is absorbed minimally by intrinsic chromophores, such as hemoglobin (<650 nm) and water (>900 nm), and thus result in noninvasive live animal imaging. Fluorescence imaging using NIR fluorescent agents displays relatively low autofluorescence. Immediately after intravenous injection, HspG41C-CTT showed continuous accumulation in tumors, and NIR fluorescence intensities reached a maximum at 3 h post-injection (Figure 5a,b). However, in HT29 tumor-bearing mice as the negative control, HspG41C-CTT showed less accumulation in tumors. Examination of tissue sections obtained at 3 h post-injection revealed HspG41C-CTT nanocages in HT1080 tumor tissues (Figure 5c), whereas non-targeted HspG41C nanocages had accumulated in both types of tumors (data not shown). The distributions of HspG41C after intravenous injection were similar to a previous report of accumulation in the liver and rapid excretion through kidney [28].

Considerable uptake of HspG41C-CTT in the liver, kidney and spleen suggested that the nanocages might be partially targeted to MMP-2. The CTT peptide is highly lipophilic. Lipophilic compounds are bound non-specifically to albumin in the blood and have significant clearance through the hepatobiliary system and high liver uptake [12]. However, the detailed mechanism underlying the undesirable uptake by organs is unclear, although the CTT peptide has been widely used as a targeted therapy ligand for cancer imaging and drug delivery [10,11,12,26,27]. To avoid liver uptake, modification of the peptides with sugar molecules or a polyethylene glycol linker has been reported to increase the tumor-to-liver ratio in a mouse model [29,30]. Our data suggest that the HspG41C-CTT nanocage may be a useful nanocarrier that can selectively deliver not only NIR imaging agents for functional diagnosis of metastatic tumors but also anticancer drugs for cancer treatment.

According to our previous studies, protein nanocages have several functionalities favorable for cancer therapeutic applications. First, the use of protein nanocages, which are a special class of multimeric proteins that form symmetric protein shells surrounding an empty interior space, relies on two distinct environments, the exposed exterior and confined interior that can be genetically modified and chemical conjugated, respectively [21]; Second, the container-like property of protein nanocages can be used for drug encapsulation. The targeting ability of nanocages increases their potential to serve as a drug carrier for antitumor efficacy [22]; Third, a protein engineering approach can easily achieve identical structures and chemical compositions (e.g., the number of peptide ligands) [23]. The protein nanocages modified CTT peptides may not only be suited for cancer imaging as the previous reports but also be used for delivery of cancer drugs. The protein nanocages modified CTT peptides may not only be suited for cancer imaging as the previous reports but also be used for delivery of cancer drugs. The capabilities of protein nanocages will support the translation of theranostic multifunctional nanomedicine with high contrast enhancement and potential use in targeted clinical applications.

## 3. Experimental Section

### 3.1. Protein Expression

The pET21a (+) vector encoding the HspG41C mutant, in which the Gly residue at position 41 of wild-type Hsp16.5 was substituted with a Cys residue, was prepared by polymerase chain reaction (PCR)-mediated mutagenesis using appropriate primers [21]. This HspG41C vector was used as a template to prepare a vector encoding the HspG41C-CTT recombinant protein with the addition of a CTT peptide at the *C*-terminus by PCR-mediated mutagenesis using appropriate primers. The primers for HspG41C-CTT were 5'-GAGAGCGAAATCGAAGGCCGCCCCTCAGGTTGCACCACCCACTGGGGTTTCACCCTGTGTGGTTGACCGAATTCGAGCTCCGTCGACAAGCTT-3' (forward primer), 5'-ACCCTCGGATTCGCCCTCGCTCTCGCCTTCAGATTCACCGCCAGAGCCACCACTAGTTTCAATGTTGATTCCTTTCTTAATTGAGGATTCTGC-3' (reverse primer). Successful mutagenesis was confirmed by DNA sequencing.

### 3.2. Protein Purification

*E. coli* [BL21-Gold (DE3)] transformed with pET21a (+) plasmid vectors were grown in 2xYT medium containing ampicillin (100 μg/mL) at 37 °C. Expression of the recombinant protein was induced by addition of 1 mM IPTG (Wako Pure Chemical Ind. Ltd., Osaka, Japan) when the culture reached an optical density of 0.5–0.6 at 600 nm. After 4 h of induction, the cells were collected by centrifugation and suspended in 50 mM NaH_2_PO_4_ containing 300 mM NaCl (pH 8.0). The cells were lysed by ultrasonic disruption and the supernatant was collected by centrifugation (15,000× *g*, 30 min). Recombinant proteins were purified from the supernatant using a HiLoad 26/10 Q Sepharose HP anion-exchange column (GE Healthcare, Tokyo, Japan) and a TSKgel G3000SW SEC column (Tosoh, Tokyo, Japan). Purity was confirmed by 15% SDS-PAGE analysis. Measurement of the molecular weight of protein nanocages was performed by MALDI-ToF mass spectrometric analysis using an Autoflex Speed (Bruker Daltonics Inc., Billerica, MA, USA) and sinapinic acid (Bruker Daltonics Inc.) as the matrix.

### 3.3. Size Measurements

The hydrodynamic diameters of protein nanocages (10 μM) were measured by dynamic light scattering using a Zetasizer Nano ZS Analyzer (Malvern Instruments Ltd., Malvern, UK) at a detection angle of 173° and temperature of 25 °C. A He-Ne laser (633 nm) was used as the incident beam. All samples and buffer solutions were filtered through an Ultrafree-MC with a pore size of 0.22 μm (Merck Millipore, Billerica, MA, USA) before measurements.

### 3.4. MMP-Binding Assay

The AlphaScreen assay (PerkinElmer Inc., Waltham, MA, USA) was performed in triplicate using 384-well white opaque plates as follows. First, 1 μL of 150 nM (3 nM final concentration) nanocages was added to each well, followed by 4 μL MMP2-Histag (Abcam, Cambridge, UK). After 2 h of incubation at 37 °C, 10 μL of a 1:100 dilution (in 0.1% bovine serum albumin/PBS) of Histag-acceptor beads was added to each well, followed by incubation for 1 h at room temperature. Then, 35 μL of a 1:62.5 dilution of donor beads conjugated rabbit-polyclonal HspG41C antibody was added to each well for a final sample volume of 50 μL. The mixture was incubated at room temperature for 1.5 h. The assay was then analyzed on an EnSpire 2300 Multilabel reader (PerkinElmer Inc., Waltham, MA, USA).

### 3.5. Conjugation of Fluorophores to Protein Nanocages

The protein nanocages and Alexa Fluor 488-maleimide or Alexa Fluor 750-maleimide (Alexa750; 1.2 eq. to protein monomer) (Invitrogen, Carlsbad, CA, USA) were incubated in phosphate buffer for 24 h at 4 °C. Unreacted fluorophore was removed by ultrafiltration using a Zeba spin column (Merck Millipore, Billerica, MA, USA).

### 3.6. Cell Study

HT1080 and HT29 cells were cultured in Dulbecco’s modified Eagle’s medium (DMEM) and RPMI-1640 medium, respectively, supplemented with 10% fetal bovine serum, 100 U/mL penicillin, 100 μg/mL streptomycin, and 0.25 μg/mL amphotericin-B (all purchased from Gibco, Grand Island, NY, USA) at 37 °C in a humidified atmosphere with 5%. To evaluate cytotoxicity, the cells were seeded in 96-well plates at an initial cell density of 1 × 10^4^ cells/well and cultured at 37 °C overnight. After the addition of protein nanocages, the cells were incubated at 37 °C for 24 h. Cell viability was measured using a CellTiter-Glo luminescent cell viability assay kit (Promega, Madison, WI, USA) according to the manufacture’s protocol. The luminescence intensity was measured using a Microplate Reader (ARVO MX 1420; PerkinElmer Inc., Waltham, MA, USA). To investigate cellular uptake, flow cytometry was performed on an ec800 (Sony, Tokyo, Japan). Cells were seeded in 6-well plates at an initial cell density of 5 × 10^4^ cells/well and cultured at 37 °C overnight. After the addition of protein nanocages labeled with Alexa Fluor 488 (1 μM), the cells were incubated at 37 °C for 3 h. After harvesting, the cells were washed once with DMEM and then resuspended in PBS. Ten-thousand cells were analyzed on the flow cytometer. Cellular uptake was assayed by excitation of Alexa Fluor 488 at 492 nm and detection of emission at 520 nm.

### 3.7. Biodistribution in Vivo

Male Balb/c mice (KBT Oriental, Ltd., Saga, Japan) were used in all experiments. Five-week-old mice were maintained in a 12-h light/dark cycle and provided with drinking water and food *ad libitum*. Animal experiments were performed according to the guidelines of the Animal Care and Use Committee, Kyushu University. HT1080 or HT29 cells (2 × 10^6^) in 100 μL Matrigel (BD Biosciences, San Jose, CA, USA) were injected subcutaneously into the right sides of the femurs of mice (18–23 g body weight). After 1–2 weeks, the tumors had reached 5–10 mm in diameter. The mice were then anesthetized, and fluorophore-labeled protein nanocages (20 μM, 250 μL) were injected intravenously into the mice. To detect nanocages labeled with Alexa Fluor 750, images were typically collected on an IVIS Spectrum (PerkinElmer Inc., Waltham, MA, USA). The IVIS excited and detected light at wavelengths of 750 and 800 nm, respectively. To detect nanocages labeled with Alexa Fluor 488, organs were dissected after intravenous injection. Staining was performed on fixed, paraffin-embedded sections of tumors in the imaging plane. The sections were then visualized under a fluorescence microscope (BioRevo BZ-9000; Keyence, Osaka, Japan).

## 4. Conclusions

In the present study, we demonstrated that systemically administered protein nanocages accumulate in tumor tissues of mice. Based on noninvasive NIR fluorescence imaging, this tumor-specific targeting ability of our protein nanocages can be explained by CTT peptide-mediated binding to metastatic cancer cells. The genetic engineering strategy used here is effective for production of nanocages that are directed to and taken up by specific cell types, target organs, or cancer cells. Ligand-mediated active binding to sites and cellular uptake are particularly valuable for the delivery of therapeutic and imaging agents that are not easily taken up by cells.
